# Molecular Genetic Mechanisms in Age-Related Macular Degeneration

**DOI:** 10.3390/genes13071233

**Published:** 2022-07-12

**Authors:** Aumer Shughoury, Duriye Damla Sevgi, Thomas A. Ciulla

**Affiliations:** 1 Department of Ophthalmology, Eugene and Marilyn Glick Eye Institute, Indiana University School of Medicine, Indianapolis, IN 46202, USA; ashughou@iu.edu (A.S.); dsevgi@iu.edu (D.D.S.); 2 Clearside Biomedical, Inc., Alpharetta, GA 30005, USA; 3 Midwest Eye Institute, Indianapolis, IN 46290, USA

**Keywords:** age-related macular degeneration, AMD, genetics, CFH, ARMS2, HTRA1, TIMP-3, gene therapy

## Abstract

Age-related macular degeneration (AMD) is among the leading causes of irreversible blindness worldwide. In addition to environmental risk factors, such as tobacco use and diet, genetic background has long been established as a major risk factor for the development of AMD. However, our ability to predict disease risk and personalize treatment remains limited by our nascent understanding of the molecular mechanisms underlying AMD pathogenesis. Research into the molecular genetics of AMD over the past two decades has uncovered 52 independent gene variants and 34 independent loci that are implicated in the development of AMD, accounting for over half of the genetic risk. This research has helped delineate at least five major pathways that may be disrupted in the pathogenesis of AMD: the complement system, extracellular matrix remodeling, lipid metabolism, angiogenesis, and oxidative stress response. This review surveys our current understanding of each of these disease mechanisms, in turn, along with their associated pathogenic gene variants. Continued research into the molecular genetics of AMD holds great promise for the development of precision-targeted, personalized therapies that bring us closer to a cure for this debilitating disease.

## 1. Introduction

Age-related macular degeneration (AMD) is one of the most common causes of irreversible blindness, affecting up to 10% of individuals over the age of 45 worldwide [1]. Progressive central vision loss due to AMD has a significant impact on patient quality of life [2,3] and is associated with substantial psychosocial [2,3,4,5] and economic [6] burden. As the global population ages over the next two decades, AMD is projected to affect up to 288 million individuals [1] and prevalence of AMD in the USA is expected to double [7].

**Figure 1:**
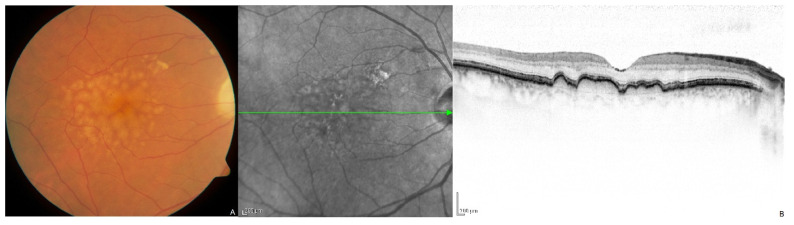
(**A**) Color fundus photo of macular drusen in the right eye of a 70-year-old male. (**B**) Optical coherence tomography image of macular drusen demonstrating sub-retinal pigment epithelium deposits. [Adapted with permission from Ipoliker, CC BY-SA 3.0 https://creativecommons.org/licenses/by-sa/3.0, accessed on 15 May 2022, via Wikimedia Commons].

AMD is characterized by the macular accumulation of sub-retinal pigment epithelial (RPE) and/or subretinal deposits known as drusen or reticular pseudodrusen, respectively, typically beginning after the seventh decade of life (Figure 1). This leads to progressive degeneration of the RPE, which plays critical metabolic and regulatory roles in supporting the health of overlying photoreceptors [8,9]. Local photoreceptor degeneration ensues, with consequent progressive loss of central visual acuity. 

Several pathophysiologic mechanisms have been implicated in the development of AMD, all of which converge on RPE dysfunction and degeneration. Oxidative stress appears to play a strong role [10]. Cigarette smoking—which increases oxidant load and impairs antioxidant defense mechanisms [11,12]—has been therefore identified as the most significant modifiable risk factor influencing the development of AMD [13]. Conversely, increased dietary and supplemental intake of antioxidant vitamins and minerals is one of the only interventions proven to slow the progression to advanced forms of disease [14]. Cumulative oxidative damage with age may cause structural degeneration of the choriocapillaris, resulting in decreased blood flow to the RPE and photoreceptors [15]. Impaired circulation reduces the clearance of lipids and cellular byproducts, which accumulate as drusen [16]. In turn, lipid accumulation can induce remodeling of the extracellular matrix (ECM) and stimulate an inflammatory response. Complex interplay between these pathogenic processes [17] ultimately causes disease progression to either tissue atrophy or macular neovascularization (MNV, formerly known as choroidal neovascularization [18]). These two endpoints represent the two major forms of advanced AMD: geographic atrophy (GA, traditionally known as “dry” AMD) and neovascular AMD (traditionally known as “wet” AMD). However, the advent of optical coherence tomography (OCT) angiography has allowed cases of nonexudative neovascularization to be more readily identified, blurring the distinction between these traditional categories [19].

Age is the strongest demographic risk factor for the development of AMD [13,14,20]. Environmental and lifestyle factors, such as cigarette smoking [12,13,21,22,23] and diet [24,25] can modify this risk. Over the past two decades, the contribution of specific genetic factors to AMD risk has been increasingly recognized. 

The genetic influence on AMD risk has long been suspected [26]. Historically, evidence for the genetic contribution to AMD came from epidemiological studies of racial disparities [27,28] and twin–twin concordance studies [29,30,31,32,33]. Over the course of history, millions of mutations in the human genome have accumulated, resulting in genetic variations such as single nucleotide polymorphisms (SNPs). These single base changes in the DNA sequence occur relatively frequently (>1 percent) and have been the subject of much study [34,35,36,37,38,39,40], especially prior to the wide availability of whole genome sequencing. In AMD, familial-based linkage [41,42,43,44,45] and genome-wide association studies (GWAS) [46,47,48,49,50,51,52] have rapidly uncovered a large number of common and rare disease-associated gene variants [53]. Such genetic research has increased our understanding of this complex disease [17,54,55] and spurred the development of novel gene-based therapies for the treatment of AMD [56,57]. 

This review surveys the literature to date implicating genetic factors in the pathogenesis of AMD. Genes identified as contributing to AMD risk are largely associated with the major pathophysiological mechanisms underlying the development of AMD: immune dysregulation and inflammation, ECM disruption, lipid accumulation, angiogenesis, and cellular apoptosis. In what follows, we discuss the major gene variants to date that have been associated with each of these pathways.

## 2. Immune Dysregulation and the Complement System

The innate immune system appears to play a key role in the development and progression of AMD. For example, immune complex deposition has been strongly implicated in the formation and biomolecular makeup of drusen [58]. It has been hypothesized that drusen may be biomarkers of immune-mediated processes occurring at the RPE–Bruch’s Membrane (BrM) interface in the aging retina [59]. Moreover, localized inflammation and microglial cell recruitment appear to be key mediators of AMD pathogenesis [54]. 

**Figure 2:**
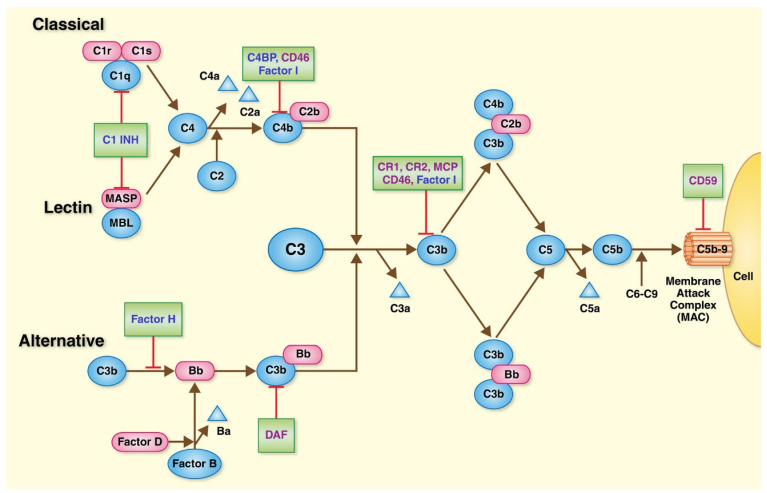
Overview of the complement system. The complement system may be activated by three distinct biomolecular pathways: classical, lectin, and alternative pathways. These three pathways converge on the cleavage of C3 to C3b, which in turn acts to cleave C5 to C5b. C5b subsequently combines with C6-C9 to form the membrane attack complex (MAC), which forms pores in the target cell membranes, resulting in lysis of the target cells. Image adapted with permission from Danobeitia et al., 2014 [65].

Dysregulation of the complement system (Figure 2) has been strongly associated with the development of AMD [58,60] and the most commonly identified high-risk genetic variants involve genes coding for key components of the complement system [51,54]. The complement cascade consists of an array of specialized plasma proteins and enzymes (complement factors) that react with one another in complex patterns to target foreign pathogens and tag them for destruction by immunologic phagocytosis [61]. Three distinct pathways have been characterized: an antibody-dependent ‘classical’ pathway, an antibody-independent ‘alternative’ pathway, and a ‘lectin’ pathway, which involves binding to specific sugars on the surface of microorganisms. All of these pathways trigger a localized inflammatory response when activated via convergence on complement factor 3 (C3), which ultimately leads to cleavage of complement factor 5 (C5) to form key terminal fragments (C5a and C5b). C5b is involved in the formation of a membrane attack complex (MAC), along with C6, C7, C8, and C9 complement factors. MAC, in turn, disrupts the lipid bilayer that forms the extracellular membrane of invading pathogens, resulting in cell lysis [61]. At sub-lytic concentrations, MAC can also induce several localized inflammatory reactions [62,63,64]. Importantly, the regulatory factors involved in these complement pathways limit complement activation specifically to the pathogenic surface [61].

### 2.1. Complement Factor H (CFH)

The complement factor H (*CFH*) gene on chromosome 1 was the earliest gene found to play a role in the development of AMD [66,67,68,69,70]. *CFH* has since been recognized as the major susceptibility locus for the development of AMD [71] in Caucasians and Asians [72,73], and CFH is deposited in significant concentrations in drusen [67,74,75]. While the exact mechanism by which CFH dysfunction confers AMD risk has not yet been established, the highest-risk CFH variants tend to affect the functional domains or serum expression of the enzyme [76]. CFH is a complement regulatory protein expressed in the BrM and sub-retinal space [77]. In the complement cascade, it functions to inhibit the alternative pathway by acting as a cofactor to complement factor I (CFI) in the inactivation of complement component 3b (C3b) [78]. CFH additionally acts to promote the decay of C3 convertase [79]. Consequently, compromise of CFH function leads to dysregulation of the complement cascade [80]. CFH also downregulates proinflammatory activity by binding to C-reactive protein (CRP) [81,82] and the immune checkpoint molecule CD47 [83]. Finally, recent functional studies have suggested that in vitro knockdown of CFH results in increased MAC deposition in choroidal endothelial cells (CECs), while overexpression of CFH protects against MAC deposition [84]. Thus, CFH dysfunction may lead to the development of localized chronic inflammation in the retina [85]. Beyond its immunomodulatory roles, it has also been suggested that CFH may independently act as a protective antioxidant in the RPE [86,87] and play key roles in lipid metabolism [88,89]. CFH involvement in multiple pathogenic pathways may help explain its strong association with AMD. 

*CFH* gene variants have been found to largely affect the functional activity of CFH, rather than serum levels [90]. Two common loss-of-function *CFH* variants—rs1061170 (Y402H) [66,67,68,69] and rs1410996 [91]—account for a significant portion of AMD genetic risk across populations [42,92,93,94,95,96,97,98,99]. These two variants are notably found at rates nearly seven-fold higher in European compared to Asian populations [100], potentially accounting for some of the difference in AMD prevalence between these two populations. The mechanism by which these variants increase AMD risk has not yet been established, although the Y402H variant has been associated with uncontrolled complement system activation [80,101]. Interestingly, although the Y402H variant is associated with a poor response to antioxidant therapy in non-neovascular AMD [98], it is associated with a strong positive response to anti-VEGF therapy in neovascular AMD [102].

A number of other rare CFH variants have been identified [42,95,101,103,104,105], many of which are highly penetrant and correlate with more severe or extensive disease phenotypes [45,106,107,108,109]. For example, the rs121913059 (p.Arg1210Cys) mutation has been found to confer a more than 20-fold increase in AMD risk [45]. 

In addition to significantly increasing the risk of AMD in general [110], *CFH* mutations have also been associated with a higher risk of particular AMD phenotypes compared to other gene variants. For example, some *CFH* mutations have been associated with a slightly stronger risk of progression to GA as opposed to MNV [54]. Conversely, *CFH* mutations may also increase the genetic risk of progression to MNV after zinc and antioxidant supplementation, as found in AREDS vitamin formulations [44,111,112,113]. Certain *CFH* variants, such as the Y402H and rs1410966 variants, have been associated with a higher risk of peripheral retinal involvement [114]. Additionally, compared to the common rs1061170 variant, rare *CFH* variants rs800292, rs1410996, and rs1329428 are associated with a relatively poor response to anti-VEGF therapy [102]. As with Y402H, many of these rare variants exhibit differences in ethnic distribution and may account for differences in AMD phenotypes across populations [115].

### 2.2. Complement Factor I (CFI)

The *CFI* gene on chromosome 4 has also been implicated in AMD. CFI functions in the alternative complement pathway to inactivate C3b in conjunction with CFH. Rare gene variants affecting CFI expression [116,117,118,119,120] and/or disrupting its functional domain [44,117,119,121] impair its ability to modulate complement activation and have been highly associated with the development of AMD [67,117]. Such variants include the p.Gly119Arg [117] and p.Leu131Arg [121] missense mutations, both of which have been associated with reduced CFI concentration and activity. An additional variant, rs915370426 (p.Pro553Ser), appears to confer high risk despite normal serum CFI concentration [121]. An investigational gene therapy, GT005 (Gyroscope Therapeutics) [122], has recently been developed to increase intraocular CFI expression in the treatment of GA. Three Phase I/II multicenter, randomized, controlled trials (“FOCUS”, NCT03846193; “EXPLORE”, NCT04437368; and “HORIZON”, NCT04566445) are currently evaluating the safety of subretinal administration of GT005 in patients with GA. Preliminary data have suggested a positive safety profile and promising evidence of a sustained increase in intravitreal CFI levels following treatment.

### 2.3. Complement Component 3 (C3)

Several rare variants in the complement *C3* gene on chromosome 19 have also been identified with an increased risk of AMD. The most common variants include rs2230199 (p.Arg102Gly) [123,124,125] and the associated polymorphism rs1047286 (p.Pro292Leu) [124,125]. These variants are particularly common in Caucasian populations and rare in Asian and African populations [54,124,125]. The rs2230199 variant is associated with impaired CFH binding, conferring resistance to CFH-mediated inactivation [126]. It has also been shown to more efficiently activate the alternative complement pathway [126]. An additional rare variant, rs147859257 (p.Lys155Gln), has been found to impair the inactivation of C3b by the CFI/CFH complex, resulting in constitutive activation of the alternative cascade pathway and a significantly increased risk of AMD [43,44,45]. Two further *C3* variants, rs117793540 (p.Arg735Trp) and rs2230210 (p.Ser1619Arg), have been inconsistently associated with AMD risk, with differing results across cohort studies [44,127]. Finally, a recent meta-analysis found no association between the two other known major *C3* polymorphisms—rs2230205, rs2250656—and AMD [125]. In fact, the rs2250656 variant may have a protective effect against the development of neovascular AMD in the Chinese population [128]. A randomized phase 2 trial investigating intravitreal injection of the C3 inhibitor Pegcetacoplan for the treatment of GA has recently published positive results, with data suggesting statistically significant effects on the progression of GA [129]; two phase 3 trials are currently underway, with GA area growth at 12 months showing a statistically significant reduction in one study (“Oaks” NCT03525613) and a trend of reduction in the other (“Derby”, NCT03525600).

### 2.4. Complement Component 5 (C5)

Few studies have evaluated the association between AMD and complement component 5 (C5). C5 is cleaved into its bioactive fragment in one of the final steps of the complement cascade (Figure 2). The role of C5 in AMD has been suggested by its presence in drusen [130,131] as well as the observation of elevated serum C5a levels in AMD [132]. It has also been suggested that C5a can induce VEGF expression, leading to the development of MNV [133]. MNV mouse models have also demonstrated that RPE and choroidal C5a levels are elevated in laser-induced MNV, while genetic knockout or pharmacologic blockade of C5a receptors reduces VEGF expression and MNV formation after laser injury [133]. 

To date, most human population studies have not demonstrated a significant association between known *C5* SNPs and AMD [123,124,134,135]. However, the prominent role of C5 in the complement cascade makes it a prime target for pharmacologic downregulation of the complement system in the treatment of AMD. A recent phase 3 study (“Gather 1” NCT02686658) demonstrated that intravitreal injection of avacincaptad pegol, a C5 inhibitor, reduced GA enlargement versus sham over a 12-month period [136]. Another confirmatory phase 3 study is underway (“Gather 2” NCT04435366). Further research is required to clarify the exact role of C5 in AMD and to uncover genetic variants that may impact risk.

### 2.5. Complement Component 9 (C9)

Recently, variants in the gene coding complement component 9 (C9) have been associated with a significantly increased risk of AMD [44,51,121,137] and progression to more advanced stages of the disease [137,138]. C9 is a key protein in the terminal complement pathway, joining with C5–C8 to form MAC, as discussed above [61]. Patients with advanced AMD exhibit higher serum MAC concentrations [139]. The rare variant p.Pro167Ser (rs34882957) has been significantly associated with AMD [44,139] and appears to be associated with an increased serum concentration of C9 [121], as well as with increased polymerization rates [139,140] and hemolytic function [139]. The *C9* p.Pro167Ser variant may therefore represent a gain-of-function mutation, leading to increased MAC formation and lytic activity in the terminal complement pathway. Other rare *C9* variants, such as p.Met45Leu, p.Phe62Ser, and p.Ala529Thr, have been associated with increased C9 expression in AMD patients, though without an increase in lytic activity [140], while other variants such as p.Arg118Trp and p.Thr170Ile have been found to confer risk without elevated C9 levels [140]. Conversely, the nonsense C9 mutation p.Arg95* found commonly in Japanese populations [141] confers a strong (nearly 5-fold) protective effect against the development of AMD [142] and has also been correlated with decreased VEGF levels and reduced risk of progression to MNV [142].

### 2.6. Complement Component 2 (C2) and Complement Factor B (CFB)

Rare variants in the *complement component 2* (*C2*) and *complement factor B* (*CFB*) genes on chromosome 6 have also been associated with a strong protective effect against the development of AMD [143,144,145,146,147]. C2 and CFB are expressed in the neural retina, Bruch’s membrane, and choroid [143], and function as activators of the classical and alternative complement cascades, respectively [148]. Variants reducing the function of these enzymes therefore dampen the activity of the complement cascade [149], potentially explaining their protective effects against the development of AMD. Such variants include *C2* rs9332739, rs547154, and rs429608, as well as *CFB* rs9332739, rs547154, rs4151667, and rs641153 [93,145,146,147,150]. Two 2012 meta-analyses found that these variants may reduce the risk of AMD by nearly half [146,147]. In the Japanese population, *C2* rs547154 and *CFB* rs541862 additionally protect against the development of MNV [151]. Interestingly, antioxidant supplementation has also been found to be more effective in retarding the progression of non-exudative AMD in patients with these protective *C2* and *CFB* variants [98].

### 2.7. Complement Factor D (CFD)

Finally, complement factor D (CFD) has also been implicated in the development of AMD. CFD functions as a rate-limiting enzyme in the activation of the alternative complement pathway by cleaving and activating CFB [152,153]. Six *CFD* variants have been identified (rs1683564, rs35186399, rs1683563, rs3826945, rs34337649, and rs1651896). These variants have largely not been significantly associated with AMD [154]. A small case-control series did find rs3826945 to be correlated with increased AMD risk [153], however this association could not be demonstrated in a separate population [93]. Interestingly, intravitreal injection of lampalizumab, a selective CFD inhibitor, was not found to reduce GA enlargement versus sham during 48 weeks of treatment in two Phase 3 trials (“Chroma” and “Spectri,” NCT02247479 and NCT02247531) [155].

## 3. Extracellular Matrix (ECM) Remodeling 

The ECM is a supportive framework consisting of the stroma and basement membrane between the epithelial and endothelial tissue layers. The retinal ECM consists of a five-layered BrM, bridging the space between the choroid and RPE [156]. It plays a crucial role in the physical support and remodeling of the RPE, as well as the exchange of biomolecules, oxygen, nutrients, and waste products between the RPE and choriocapillaris [157]. The structure of BrM is controlled by a balance between local proteolytic enzymes known as matrix metalloproteinases (MMPs) and their tissue inhibitors (TIMPs) [158]. Specifically, MMP-1, MMP-2, MMP-3, MMP-9, TIMP-1, TIMP-2, and TIMP-3 are found at high concentrations in BrM and are critical to maintaining its structure and function [159]. 

Changes in the structure and function of BrM have been strongly implicated in the pathogenesis of AMD [160]. Impaired MMP-mediated ECM degradation has been associated with both aging and macular degeneration, resulting in thickening of BrM [161]. As BrM increases in thickness, its filtration capacity declines, leading to the focal accumulation of waste products and the formation of drusen [157]. Additionally, reduced permeability of BrM impairs the transport of critical metabolites and nutrients between the RPE and choroid, ultimately leading to RPE and photoreceptor degeneration [162]. It is therefore hypothesized that an imbalance between MMP and TIMP activity may play a role in AMD pathogenesis [163]. Several gene variants affecting local MMP and TIMP expression have been associated with AMD risk [160]. 

### 3.1. Tissue Inhibitor of Metalloproteinases (TIMPs)

Variants in *TIMP-3* have been particularly strongly linked to the development of AMD [46,51,164,165]. TIMP-3 is expressed in the RPE adjacent to BrM [166] and localizes directly to the ECM [167]. In the aging retina, TIMP-3 accumulates in BrM [162,168] and particularly elevated concentrations of TIMP-3 are found in AMD [162]. As an MMP inhibitor, TIMP-3 excess results in impaired ECM turnover [169] and pathologic thickening of BrM, leading to RPE and photoreceptor atrophy [162,170]. Additionally, TIMP-3 physiologically acts as a potent local inhibitor of angiogenesis, competitively impairing VEGF binding to its receptor [171]. Decreased TIMP-3 activity thus increased retinal VEGF activity and angiogenesis. Notably, genetic variants that reduce TIMP-3 expression are the main etiology of Sorsby’s fundus dystrophy, a rare autosomal dominant macular dystrophy presenting with features of neovascular AMD at a young age [172]. 

Variants in *TIMP-3* contribute significantly to the genetic burden in patients with AMD [51]. In their 2016 GWAS, Fritsche et al. found nine rare variants in *TIMP-3* to be cumulatively associated with a >30-fold increased risk of AMD [51]. In addition to contributing to the overall risk of AMD, it is thought that TIMP-3 dysfunction or suppression may contribute to the development of MNV [173]. The rs5754227 [51], rs713685 [174], rs743751 [174], and rs5749482 [175] *TIMP-3* intron variants have been particularly associated with an increased risk of AMD. Conversely, the rs9621532 *TIMP-3* variant has been found to have a slight to moderate protective effect against AMD in some studies [47,49,165,176], as well as a protective effect against the development of MNV [177]—although other studies have found no association with AMD risk [178,179]. Finally, *TIMP-3* variants rs6518799, rs756481, rs5749498, rs12170368, and rs1427385 have not been found to be associated with AMD [174,180]. Beyond *TIMP-3*, the *TIMP-2* gene (specifically polymorphism rs8179090) has also been identified and found to be inconsistently associated with decreased AMD risk [181,182].

### 3.2. Matrix Metalloproteinases (MMPs)

Several studies have also demonstrated an association between MMP gene mutations and AMD. *MMP-2* is currently the most widely studied gene in this context. Cheng et al. demonstrated that the T allele (TT and CT genotypes) of the rs243865 *MMP-2* polymorphism is protective against AMD [173], while Liutkeviciene et al. found an association between the homozygous CC genotype and hard drusen development in AMD [183]. Conversely, several studies [181,184,185,186] including a recent meta-analysis [187] have demonstrated no statistically significant association between the *MMP-2* rs243865 variant and AMD. Beyond rs243865, the *MMP-2* rs2287074 variant has been found to have a protective effect against AMD in one study [186], while the rs243866 and rs2285053 variants have not demonstrated an association with AMD [182,184,188]. 

The *MMP-9* rs142450006 [51,189,190], rs3918241 [182], rs3918242 [182,184], rs4810482 [190], rs17576 [190], and rs17577 [190] variants are associated with an increased risk of AMD and progression to MNV. Similarly, the *MMP-9* CA (13–27) microsatellite expansion variant has also been associated with progression to MNV, with risk directly proportional to the length of microsatellite expansion [191]. 

No association has been found between the known variants in *MMP-1* (rs1799756) [192], *MMP-3* (rs3025058) [184,193], or *MMP-7* (rs11568818) [182,192] and AMD. 

### 3.3. Other Extracellular Matrix Components

Other genes coding for ECM components have been studied in association with AMD. Collagen Type 8, α 1 (COL8A1), is a short-chain component of type VIII collagen found in the basement membrane of many components of the human eye [194], including BrM [195]. A common gene variant, rs140647181, near the *COLA8A1* gene has been associated with increased AMD risk [51]. A wide array of additional rare, protein-altering variants in the *COL8A1* gene itself has also been independently associated with AMD [195,196]. Similarly, the rs1999930 gene variant near the collagen matrix protein-coding genes *COL10A1* (coding for α chain of type X collagen) and *FRK* (coding for fyn-related kinase) have also been associated with increased AMD risk [48], as they have copy number variants in the *EFEMP1* gene coding for fibulin 3, a matrix glycoprotein [197]. The mechanism by which many of these gene alterations influence AMD risk remains unclear, although it is suspected that these variants may alter the integrity of BrM [195] or cause ECM protein accumulation in BrM as drusen [198]. 

## 4. Lipid Metabolism

**Figure 3:**
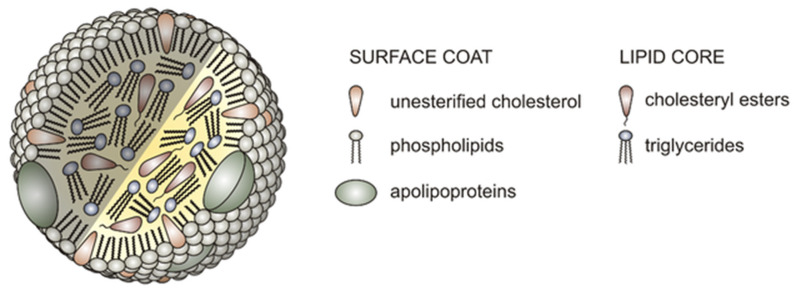
Structure of a lipoprotein. Lipoproteins are biochemical structures that function in the transport of water-insoluble lipids in the bloodstream. They consist of a hydrophilic outer shell (surface coat) composed of apolipoproteins, phospholipids, and unesterified cholesterol, and a lipid core consisting of cholesteryl esters and triglycerides. (Image adapted with permission from: AntiSense, CC BY-SA 3.0, https://creativecommons.org/licenses/by-sa/3.0, accessed on 15 May 2022, via Wikimedia Commons).

Lipid accumulation in BrM is strongly implicated in AMD pathogenesis [199,200]. Much like the intima of atherosclerotic arterial walls, lipids, and cholesterol accumulate significantly in human BrM with age [201], causing pathologic thickening and dysfunction of BrM [202]. Additionally, the RPE basolaterally secretes large lipoproteins (Figure 3) containing apolipoproteins B and E into BrM as a byproduct of photoreception [200]. In AMD, the reduced clearance of lipids from BrM may be a key mechanism in the formation of drusen [200,203]. Lipids are among the most significant components of drusen [204,205,206], accounting for over 40% of the drusen volume [205]. Large lipoproteins containing apolipoproteins B and E are secreted basolaterally by the RPE into BrM; these accumulate in both the sub-RPE space as soft drusen, as well as in the subretinal space as drusenoid deposits (also known as “pseudodrusen”) [207]. Pathologic accumulation of lipids in these spaces leads directly to RPE and photoreceptor loss in AMD [208] as in the critical flow of biomolecules across the BrM between the RPE-photoreceptor complex and choriocapillaris is impaired [203]. Drusen expansion additionally disrupts the local cellular architecture by driving RPE cells into the retina, resulting in RPE degeneration and atrophy [203]. Moreover, as in atherosclerotic disease [209], oxidation of lipoproteins in BrM and the local inflammatory response they induce [210] likely also play a key role in the pathogenesis and progression of AMD [59]. 

**Figure 4:**
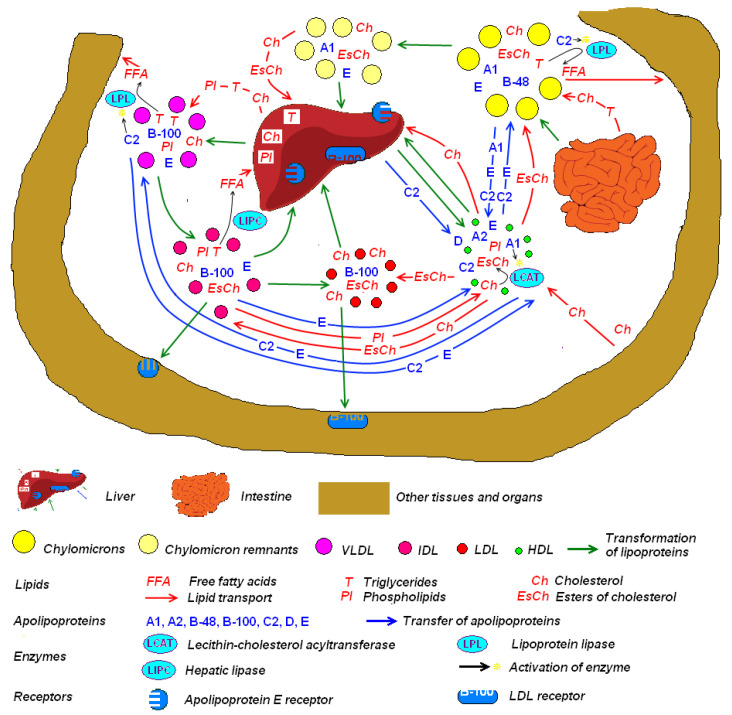
Lipid transport and metabolism in the human body. Apolipoproteins (e.g., A1, A2, B-48, B-100, C2, D, and E) bind water-insoluble lipids to form water-soluble lipoproteins such as chylomicrons, very low-density lipoproteins (VLDL), low-density lipoprotein (LDL), and high-density lipoprotein (HDL) to allow the transport of lipids (e.g., cholesterol, triglycerides) through the bloodstream. The process of lipoprotein formation is mediated by enzymes such as lecithin-cholesterol acyltransferase (LCAT). Upon arrival in their target tissues, lipids carried by lipoproteins are broken down by enzymes such as lipoprotein lipase (LPL) in peripheral tissue and hepatic lipase (LIPC) in the liver. (Image adapted with permission from: Vtosha, CC BY-SA 3.0, https://creativecommons.org/licenses/by-sa/3.0, accessed on 15 May 2022, via Wikimedia Commons).

Variants in several genes coding for proteins involved in lipid metabolism and cholesterol transport (Figure 4) are associated with the risk of AMD. Genes involved in the structure and function of high-density lipoprotein (HDL) are particularly implicated. HDL functions in the removal and transport of excess cholesterol from peripheral tissues to the liver [211]. HDL also exerts localized anti-inflammatory effects by inhibiting monocyte activity [212] and is a major transporter of lutein and zeaxanthin in the retina [213]. 

### 4.1. Apolipoprotein E (ApoE)

Lipoproteins function in the human body as transport vehicles for water-insoluble cholesterol. In particular, HDL-mediated transport of cholesterol to the liver is the main mechanism by which peripheral tissues recycle excess cholesterol [211]. The formation and metabolism of such plasma lipoproteins is regulated by associated apolipoproteins [214]. Apolipoprotein E (ApoE) is significantly involved in regulating cholesterol metabolism and lipid transport in nervous tissue [215]. ApoE dysfunction has therefore notably been found to play a key role in the pathogenesis of Alzheimer’s disease and other neurodegenerative disorders [Huang 2014]. It is also a major component of drusen [215]. ApoE has consequently been a particular focus of AMD research. 

The *APOE* gene is among the earliest genes associated with AMD [41]. Three major allelic variants, *ApoE2*, *ApoE3*, and *ApoE4*, have historically been identified as giving rise to structurally and functionally distinct protein products [216]. The *ApoE3* allele is the most common, occurring in more than 75% of chromosomes worldwide [217], and it is considered the wild-type, “neutral” allele. The *ApoE4* allele, with an estimated frequency of 15% worldwide [218], is associated with a strong protective effect against AMD [41,100,219,220,221,222,223,224,225,226]. A 2006 meta-analysis estimated that the *ApoE4* allele confers up to a 40% reduction in the risk of developing AMD [100]. On the other hand, *ApoE2*, with an estimated frequency of 8% worldwide [217], has been associated with a slightly increased risk of AMD in several independent studies [222,223,227,228,229], although a recent large meta-analysis did not find any association between *ApoE2* and AMD [224]. A large study of a Brazilian population failed to find an association between *ApoE2* and AMD [225]. However, *ApoE2* may play a role in the pathogenesis of MNV by upregulating angiogenic factors [230].

### 4.2. Hepatic Lipase (LIPC)

Plasma HDL levels are strongly regulated by hepatic lipase (LIPC) [231], which is expressed in the subretinal space and involved in intra-retinal lipid transport [232]. Several studies have demonstrated a protective effect of *LIPC* variants (rs493258, rs10468017, rs9621532, rs11755724, rs493258, rs509859, rs12637095) against the development of AMD [47,48,233,234,235,236,237]. Two of these *LIPC* variants—rs493258 and rs10468017—have been specifically associated with increased systemic HDL levels [238]. However, the mechanism by which these variants reduce AMD risk is unclear, as elevated HDL levels alone do not appear to account for the observed risk reduction [47,233]. In fact, elevated HDL levels may instead be associated with an increased risk of AMD [60]. It has been suggested that these variants may increase the HDL-mediated efficiency of carotenoid delivery to the retina [47]. The rare *LIPC* rs13095226 and rs3748391 variants have also been associated with a slightly increased AMD risk [47]. Finally, *LIPC* variants may be associated with a poorer response to anti-VEGF therapy [239].

### 4.3. Cholesteryl Ester Transfer Protein (CETP)

Cholesteryl ester transfer protein (CETP) has been strongly associated with AMD. CETP plays a key function in the transport of cholesterol from peripheral tissue to the liver by transferring cholesterol esters from low-density lipoproteins (LDLs) and very low-density lipoproteins (VLDLs) to HDLs [240]. In the human retina, CETP localizes to photoreceptor outer segments and the choriocapillaris and is involved in local lipid trafficking [241]. The major *CETP* variant, rs3764261, has been strongly associated with an increased risk of AMD [46,47,49,50,234]. Interestingly, Wang et al. noted this association only after adjusting for pathologic *CFH* variants, suggesting the presence of major interplay between the complement and lipid metabolism pathways [234]. Two additional intronic variants of *CETP* have been associated with AMD: rs5817082 is associated with a slightly reduced risk of AMD, while rs17231506 is associated with a slightly increased risk [51]. Curiously, *CETP* rs17231506 is associated with elevated HDL levels [60,242] similarly to *LIPC* rs493258, yet the two gene variants appear to have opposite effects on AMD pathogenesis. 

### 4.4. ATP-Binding Cassette Transporter A1 (ABCA1)

The ATP-binding cassette transporter A1 (ABCA1) protein plays an important role in the elimination of excess tissue cholesterol by initiating the formation of HDL [243] and mediating the efflux of cellular cholesterol into the extracellular space, where HDL may bind and transport it back to the liver [181]. The complete absence of ABCA1 in a knockout mouse model results in retinal lipid accumulation and RPE degeneration [244], whereas increased ABCA1 expression leads to reduced lipid accumulation in in-vitro RPE cell models [245]. Population-based studies of the rs1883025 *ABCA1* variant have demonstrated opposite effects of its two alleles; the C allele is generally associated with increased plasma HDL levels and increased risk of AMD, while the T allele is associated with decreased HDL and decreased risk of AMD [49,246]. The protective effect of the rs1883025 T allele has also been demonstrated in a 2016 meta-analysis [247]. Similarly, the A allele of the rs2740488 variant has been associated with an increased risk of AMD, whereas the C allele is associated with a slightly decreased risk [51,245]. 

### 4.5. Lipoprotein Lipase (LPL)

Finally, lipoprotein lipase (LPL) is an enzyme ubiquitously expressed throughout the human body with key roles in the metabolism of lipoprotein triglycerides into free fatty acids in the blood stream [248]. A single intergenic *LPL* variant, rs12678919, has been analyzed in the context of AMD, with inconsistent findings. Several studies have demonstrated no statistically significant association between rs12678919 and AMD [47,176,249,250,251]. However, two studies have demonstrated an association with increased risk [46,252]. As with *CETP*, a meta-analysis by Wang et al. discovered a strong association between *LPL* rs12678919 and AMD risk only after adjusting for *CFH* gene mutations [234], which may account for the variable findings of prior studies. 

## 5. Angiogenesis

Angiogenic pathways are involved in the development of MNV in the advanced stages of neovascular AMD. MNV involves the proliferation of new abnormal blood vessels growing from the choroid into the sub-RPE or subretinal space, or both [253]. Various hypotheses have been proposed to account for this phenomenon. It is predominantly thought that subretinal angiogenesis is controlled by the RPE [254], which secretes VEGF in states of ischemia to stimulate endothelial cell proliferation [255]. VEGF is therefore recognized as the major driver of neovascularization, and currently represents the main target for treatment of neovascular disease [256]. The RPE also secretes pigment epithelial-derived factor (PEDF), a potent inhibitor of angiogenesis [257], allowing the modulation of neovascularization via PEDF activity. It has also been suggested that certain ECM components can stimulate angiogenesis in states of dysregulation [258].

### 5.1. Vascular Endothelial Growth Factor (VEGF)

**Figure 5:**
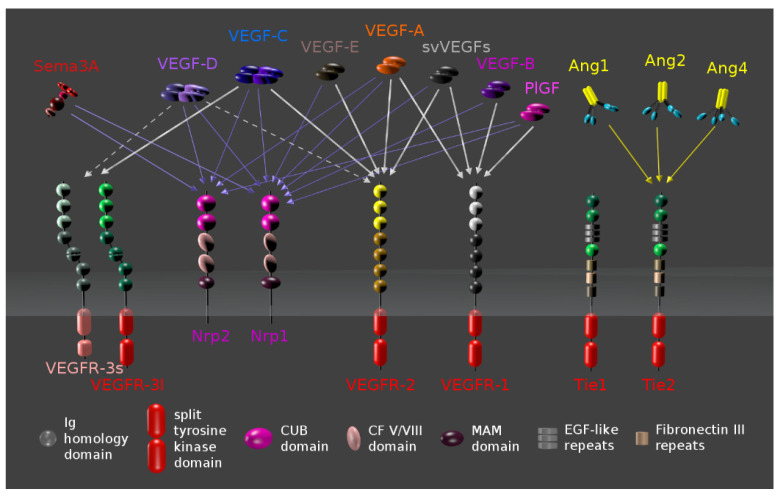
Vascular endothelial growth factor (VEGF) isoforms and their receptors (VEGFR). (Image adapted with permission from: Mjeltsch, CC BY-SA 4.0, https://creativecommons.org/licenses/by-sa/4.0, accessed on 15 May 2022, via Wikimedia Commons).

VEGF consists of seven biologically active isoforms (Figure 5), with VEGF-A playing the most significant role in angiogenesis [259]. VEGF-A is a glycoprotein that mainly targets endothelial cells to stimulate proliferation, migration, and vessel formation while inhibiting cellular apoptosis [260]. Elevated levels of VEGF-A have consequently been associated with a range of ocular neovascular diseases [261]. Multiple *VEGF-A* variants have been identified, most of which surprisingly have not been strongly or consistently associated with AMD [38,262]. However, the *VEGF-A* rs3025033 variant and haplotype rs1570360A-rs699947A-rs3025033G-rs2146323A have recently been associated with a lower risk of neovascular AMD [263]. Conversely, the *VEGF-A* rs3025039 C-allele may increase the risk of AMD, although apparently only in the context of the rs1048661 lysine oxidase 1 (*LOXL1*) gene variant [264]. The T-allele of the *VEGF-A* rs3025000 variant increases the likelihood of clinical response to anti-VEGF therapy in MNV [265], an effect that has not been demonstrated with other VEGFA alleles [266] and may be relatively minor compared to phenotypic predictors of response to VEGF therapy [267]. 

### 5.2. Fibulin 5

Fibulin 5 (FBLN5) is an ECM protein that localizes to sub-RPE deposits and drusen [268,269] and plays a role in modulating angiogenesis in part by antagonizing VEGF [270]. AMD patients have been found to secrete FBLN5 at reduced rates [271]. Missense *FBLN5* and other fibulin gene mutations can be found in up to 2% of AMD patients [272,273], suggesting that fibulin dysfunction may play a role in AMD pathogenesis [272]. 

## 6. Oxidative Stress Response and Photoreceptor Survival

The shared endpoint of the pathophysiologic processes underlying macular degeneration is RPE degeneration, with consequent photoreceptor cell death and dysfunction [274]. This process may be expedited by direct oxidative damage to cellular DNA in the retina directly causing cellular apoptosis [275]. Genetic mutations that increase susceptibility to oxidative damage and impair photoreceptor survival may therefore play a role in AMD pathogenesis [10]. For example, the *RAD51* family of genes plays a critical role in DNA repair and protection against oxidative damage [276]. Several rare mutations in *RAD51B* have been associated with a significantly increased risk of AMD [223,277,278], and abnormally decreased serum concentrations of RAD51B have been noted in AMD patients [279]. 

Similarly, the tumor necrosis factor receptor superfamily, member 10a (TNFRSF10A), also known as Death Receptor 4 (DR4), plays key roles in promoting cellular apoptosis [280] and can be localized to the RPE [281]. Reduced expression of TNFRSF10A is associated with decreased RPE cell viability and increased apoptotic susceptibility in mice [281]. *TNFRSF10A* mutations have been associated with an increased risk of AMD, particularly in Asian populations [50,51,151,282,283].

Finally, excision repair cross complexes (ERCC) have been associated with AMD susceptibility [284]. ERCC6 functions in the transcription-coupled excision repair of DNA mutations [285]. Patients with AMD have been found to have decreased retinal ERCC6 expression [286]. Mutations in the *ERCC6* gene have been inconsistently associated with increased AMD risk [179,284,286,287,288] and may have a synergic effect with *CFH* mutations [284].

## 7. Genes Implicated in Multiple Pathways

### Age-Related Maculopathy Susceptibility 2 (ARMS2) and High-Temperature Requirement Factor A Serine Peptidase 1 (HTRA1)

One of the earliest discovered and most significant of the susceptibility loci associated with AMD is the region of chromosome 10q26 spanning the age-related maculopathy susceptibility 2 (*ARMS2*) gene coding region and the high-temperature requirement factor A serine peptidase 1 (*HTRA1*) gene promoter [44,289,290,291,292,293,294]. The *ARMS2-HTRA1* region is a major susceptibility locus among Caucasians and East Asians [72,73], together with *CFH* accounting for over half of the genetic risk associated with AMD [51,153,229,295,296,297]. Notably, patients with mutations in the *ARMS2-HTRA1* locus are more than twice as likely to progress to late-stage disease compared to patients harboring *CFH* variants, and *ARMS2-HTRA1* variants have also been associated with more rapid progression of disease [17]. 

Multiple high-risk gene variants have been discovered in the *ARMS2-HTRA1* locus [95,223,294,296,298,299,300,301,302,303,304,305]. These variants are in high linkage disequilibrium (LD), and most studies to date have been unable to statistically distinguish between them. It is therefore unclear to date which of *ARMS2* or *HTRA1* is responsible for increased AMD risk [306]. Moreover, the exact structure and function of ARMS2 and HTRA1 remain unclear.

Local ARMS2 dysfunction may play a role in oxidative stress and damage to the retina [306,307]. Early studies suggested that ARMS2 localizes to the outer mitochondrial membrane in photoreceptors and RPE cells [302,307,308], although this finding has been disputed [309,310]. It has also been suggested that ARMS2 localizes to the perinuclear cytoplasm [309] or endoplasmic reticulum [310], suggesting a non-mitochondrial mechanism of increasing AMD risk. Finally, ARMS2 has recently been implicated in the local inflammatory response secreted by macrophages to activate the complement cascade and clear cellular debris [311]. HTRA1, on the other hand, has been implicated in a broad range of physiologic processes, including ECM remodeling and TGF-β cytokine signaling [306]. Its most prominent roles appear to be in ECM deposition, angiogenesis, and regulation of local subretinal inflammation [294,312,313]. It has also been hypothesized that HTRA1 plays a key role in maintaining the RPE–BrM–choroid interface in the aging retina [314]. High-risk variants that reduce HTRA1 expression at the RPE–BrM interface may therefore give rise to AMD phenotypes due to loss of its protective function against the effects of advancing age [314]. Some have recently argued that the weight of the evidence to date supports *HTRA1*, rather than *ARMS2*, as the main causal genetic factor of the two associated with AMD [306]. 

The *HTRA1* gene variant rs11200638 is found in the *HTRA1* promoter region in strong LD with the *ARMS2* missense variant rs10490924 [294,300,309]. Both have been highly associated with increased AMD risk [95,223,305,308,309,315,316,317] and a younger onset of the disease [304]. *ARMS2* rs10490924 is correlated with elevated C-reactive protein levels [318], suggesting an inflammatory mechanism at play. Interestingly, while the *ARMS2* rs10490924 G allele is associated with an increased risk of progression to advanced stages of disease [296], the G allele is also associated with a higher likelihood of MNV response to anti-VEGF therapy in patients who have progressed to neovascular disease [98,317,319,320,321,322], particularly in East Asian populations [321]. A similar correlation has been demonstrated between *HTRA1* rs11200638 and increased likelihood of response to anti-VEGF therapy [317]. The *ARMS2* rs10490924 variant also appears to play a more prominent role in AMD development in Asian populations than in European populations, with risk allele frequencies of 40% vs. 20%, respectively [54]. As ARMS2 dysfunction is slightly associated with the progression to neovascular disease, these differences in allele distribution may in part explain the higher prevalence of MNV in Asian populations compared to Europeans [323]. [

An additional unstable *ARMS2* insertion-deletion variant c.*372_815del443ins54 in complete LD with *ARMS2* rs10490924 and *HTRA1* rs11200638 has also been strongly associated with increased AMD risk and progression to MNV [95,308,324]. However, its effects on HTRA1 and ARMS2 expression and function are unclear, and the risk it confers is difficult to distinguish from that associated with rs10490924 and rs11200638 [306]. Another *ARMS-HTRA1* variant, rs2284665, has also been found to correlate significantly with the development of MNV [325]. Finally, the nonsense *ARMS2* variant rs10490924 [308] appears to have no effect on AMD risk despite decreasing ARMS2 expression [326].

In addition to *HTRA1* rs11200638, other variants, such as rs1049331, rs2293870, and rs2284665, have also been identified as strongly associated with AMD progression [327,328,329]. The mechanism by which theses *HTRA1* mutations confer risk is unclear. Impaired HTRA1-mediated inhibition of cellular apoptosis [330] and insulin-like growth factor 1 (IGF1) [331] may play a role. Additionally, *HTRA1* rs2284665 may increase HTRA1 expression in lymphocytes and elsewhere [294], although this has not been consistently demonstrated [294,332,333,334,335].

## 8. Conclusions

AMD is a complex and multifactorial disease that we have yet to fully understand. Several pathogenic pathways involving the complement system, extracellular matrix remodeling, lipid metabolism, angiogenesis, and oxidative stress response are currently thought to intertwine to give rise to AMD in its various forms. Age plays a significant role in the risk of disease development, and environmental factors such as smoking and diet represent important modifiable risk factors to prevent AMD. Despite its late onset, AMD has also been shown to have a strong genetic component, and a broad array of gene variants affecting these key pathways have been identified as significantly affecting the risk of disease. With the advent of GWAS, at least 52 independent gene variants and 34 genetic loci have been identified to date, accounting for over 50% of the genetic risk [51]. There are now commercially available tests (e.g., Macula Risk and Vita Risk^®^ from Arctic Medical Laboratories, Grand Rapids, Michigan, USA) that assess for the presence of gene variants affecting these pathways. Such testing may allow an estimation of personalized risk and expected response to treatment using algorithms that integrate genetic information with other disease-associated factors such as age, smoking status, fellow-eye status, and body mass index [336]. While the utility of genetic testing remains limited at this time due to the paucity of approved gene-targeted treatment modalities [337], gene therapies currently in development [57,338] hold great promise for the future implementation of personalized interventions that significantly alter the course of AMD. Future research on the molecular and pharmacogenetics of AMD may thus hold the key to curing what is currently among the most common incurable causes of blindness worldwide.
